# Development of in-House Synthesis and Quality Control of [^99m^Tc]Tc-PSMA-I&S

**DOI:** 10.3390/molecules28020577

**Published:** 2023-01-06

**Authors:** Elisabeth Plhak, Christopher Pichler, Edith Gößnitzer, Reingard M. Aigner, Herbert Kvaternik

**Affiliations:** 1 Division of Nuclear Medicine, Department of Radiology, Medical University of Graz, Auenbruggerplatz 9, 8036 Graz, Austria; 2 Department of Pharmaceutical Chemistry, Institute of Pharmaceutical Sciences, University of Graz, Schubertstraße 1/EG/0122, 8010 Graz, Austria

**Keywords:** [^99m^Tc]Tc-PSMA-I&S, automated preparation, technetium-99m

## Abstract

Many radioactive PSMA inhibitory substances have already been developed for PET diagnostics and therapy of prostate cancer. Because PET radionuclides and instrumentation may not be available, technetium-99 m labelled tracers can be considered as a diagnostic alternative. A suitable tracer is [^99m^Tc]Tc-PSMA-I&S, primarily developed for radio-guided surgery, which has been identified for diagnostics of prostate cancer. However, there is no commercial kit approved for the preparation of [^99m^Tc]Tc-PSMA-I&S on the market. This work presents an automated process for the synthesis of [^99m^Tc]Tc-PSMA-I&S concerning good manufacturing practice (GMP). We used a Scintomics GRP 4 V module, with the SCC software package for programming sequences for this development. The optimum reaction conditions were evaluated in preliminary experiments. The pH of the reaction solution was found to be crucial for the radiochemical yield and radiochemical purity. The validation of [^99m^Tc]Tc-PSMA-I&S (*n* = 3) achieved a stable radiochemical yield of 58.7 ± 1.5% and stable radiochemical purities of 93.0 ± 0.3%. The amount of free [^99m^Tc]TcO_4_^−^ in the solution and reduced hydrolysed [^99m^Tc]TcO_2_ was <2%. Our automated preparation of [^99m^Tc]Tc-PSMA-I&S has shown reliability and applicability in the clinical setting.

## 1. Introduction

**Figure 1:**
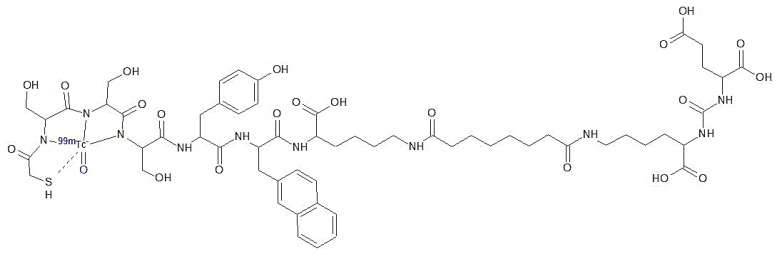
Chemical structure of [^99m^Tc]Tc-PSMA-I&S.

The prostate-specific membrane antigen (PSMA) is an essential target for the diagnosis and therapy of prostate cancer. It belongs to the membrane-type zinc peptidase family and has two functions: as a receptor, and as a zinc-protease enzyme. It is overexpressed in prostate cancer and its metastatic lesions, which makes it an exciting target for imaging and therapy of prostate cancer [1]. [^68^Ga]Ga-PSMA-11 is currently the most used radiopharmaceutical for the diagnosis of prostate cancer. However, the instrumentation and radionuclides for positron emission tomography (PET) applications are not only expensive but also of limited availability in many countries. Therefore, a lot of effort has been employed in developing PSMA targeting tracers for single-photon emission computed tomography (SPECT) [2]. [^99m^Tc]Tc-PSMA-I&S was first introduced by Robu et al., including the preclinical evaluation and first patient application. The abbreviation I&S (imaging and surgery) means that the tracer represents a dual function: It can be used for diagnostic imaging and the surgical resection of PSMA-positive lesions by using a gamma probe. The bifunctional ligand consists of a mercaptoacetyl-triserin (MAS3) chelator binding the [Tc≡O]3 + core, coupled to the PSMA-targeting peptide Lys-urea-Glu (Figure 1). [^99m^Tc]Tc-PSMA-I&S has a high in vivo stability and blood clearance is relatively slow. The best tissue-to-background ratios are reached at later time points, over 5 h after administration, and steadily increase over time due to the long availability of the stable tracer in the blood [3].

Due to its tracer kinetics, [^99m^Tc]Tc-PSMA-I&S is highly suitable for radio-guided surgery [4,5]. According to dosimetry calculations based on the half-life of technetium-99m, the activity in tumour lesions remains in a detectable range for commercially available γ-probes for up to 48 h [6]. In the current literature, the tracer application was constantly performed between 16 and 24 h before surgical treatment [3,4,5,7,8]. [^99m^Tc]Tc-PSMA-I&S has been tested in robot-assisted radio-guided surgery using a drop-in gamma probe [7,8,9]. Currently, the benefits of this procedure are being evaluated in a clinical trial. The first interim analysis of the study showed that [^99m^Tc]Tc-PSMA-I&S can help surgeons to identify and remove affected lymphnodes, but is not sensitive enough to identify micrometastatic tissue [8].

In addition the diagnostic performance of [^99m^Tc]Tc-PSMA-I&S is promising. The first study on the diagnostic use of [^99m^Tc]Tc-PSMA-I&S-SPECT/CT showed that the tracer is applicable for evaluating of biochemical recurrence, primary staging, and restaging of prostate cancer. Imaging was performed at 5 h post injection. Although significant tracer accumulation was observed in the liver, the gastrointestinal tract and urinary bladder at this time, additional low dose CT allowed good discrimination between physiological uptake and pathologic lesions. However, at low PSA levels (<4 ng/mL) the detection rate of [^99m^Tc]Tc-PSMA-I&S is inferior to [^68^Ga]Ga-PSMA-11 PET/CT, so it requires a careful patient selection if PET/CT imaging is available [10]. A dosimetry study after administration of 700 MBq [^99m^Tc]Tc-PSMA-I&S, similar to other ^99m^Tc-tracers, resulted in an average effective body dose of 3.64 mSv to healthy volunteers [6].

Due to the increasing number of patients, the Division of Nuclear Medicine Graz decided to introduce [^99m^Tc]Tc-PSMA-I&S as a possible partial substitute for [^68^Ga]Ga-PSMA-11. Furthermore, Aalbersberg et al. presented a method of producing [^99m^Tc]Tc-PSMA-I&S on a Scintomics GRP synthesizer using commercially available single-use kits for ^68^Ga-peptides [11]. Our goal was to use the free programmable GRP developer software to configure and optimise the kit setup and the automated labelling process. For instance, an additional tubing line to enter technetium-99m pertechnetate ( [^99m^Tc]TcO_4_^−^) was introduced, and the composition of the reaction mixture was optimised. In addition, the quality control was carried out similarly to gallium-68 labelled tracers and underwent a full validation.

## 2. Results

### 2.1. Automated Radiolabelling

**Figure 2:**
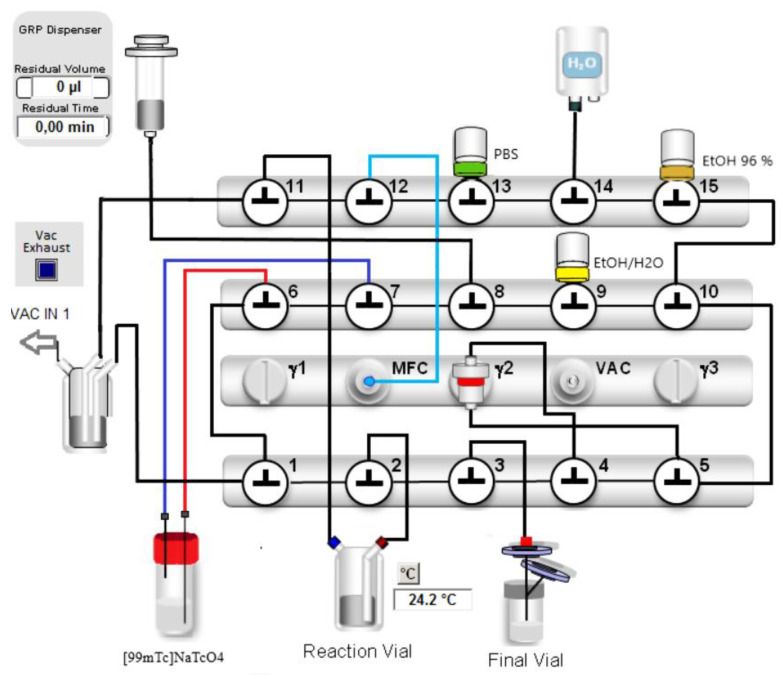
Configuration of the Scintomics GRP module for the labelling of [^99m^Tc]Tc-PSMA-I&S.

The automated synthesis was developed on a Scintomics GRP Synthesis module assembled with modified single use kits for the labelling of ^68^Ga-peptides. The configuration is shown in Figure 2. The process sequences were created with the Scintomics developer software.

In preparation for the automated process, the PSMA-I&S precursor (40 µg) was diluted in HEPES (4-(2-hydroxyethyl) piperazine-1-ethanesulfonic acid) buffer and transferred into the reaction vessel. Then, a tin (II) chloride (SnCl_2_)/ascorbic acid solution, as well as sodium hydroxide (NaOH), was added to the precursor solution. Furthermore, [^99m^Tc]TcO_4_^−^ was placed in a V-shaped vial with a lead shielding, which was connected with tubes and needles to the apparatus via valves 6 and 7.

The synthesis started with the preconditioning of the Sep-pak^®^ Light C18 cartridge with ethanol and water. During the automated process, [^99m^Tc]TcO_4_^−^ was flushed from the V-Vial into the reaction vessel with nitrogen gas (N_2_). As a result, 99.2 ± 0.2% of the starting activity was successfully transferred to the reaction solution. The labelling was performed at 105 °C within 20 min. After that, the process was likewise carried out to ^68^Ga-labelled peptides through purification of the compound over a Sep-Pak^®^ Light C18 cartridge, followed by elution with 50% ethanol and dilution with phosphate-buffered saline (PBS) via a sterile filter. The total volume of the final product was 17.0 ± 1.0 mL. The total processing time was 40 min.

### 2.2. Optimising the Reaction Conditions

The commercially available kit including the reagents and labelling cassettes was originally designed to label ^68^Ga-peptides at a pH of about 3.5–5. For the labelling of [^99m^Tc]Tc-PSMA-I&S, preliminary labelling experiments were conducted to determine the optimum reaction conditions. The pH adjustment was done through the addition of 10 M NaOH to the reaction solution. A pH of 5.5 formed a high amount of reduced hydrolysed technetium-99m ( [^99m^Tc]TcO_2_), retarded in the reaction vessel and onto the Sep-Pak^®^ Light C18 cartridge. The radiochemical yield of [^99m^Tc]Tc-PSMA-I&S was only 0.5%. A reaction pH of 7.2 raised the yield to 46%. The highest radiochemical yields were achieved with reaction conditions between pH 7.8 and 8.2 (Table 1).

**Figure 3:**
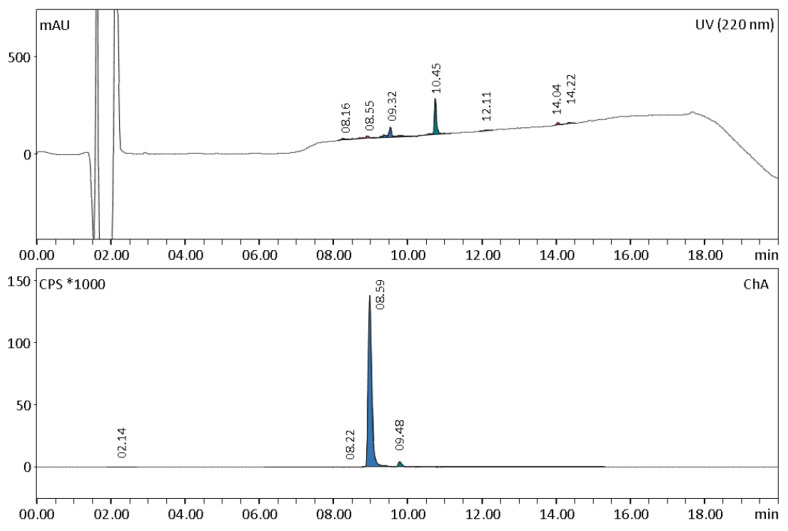
Representative radio–HPLC chromatogram of a [^99m^Tc]Tc-PSMA-I&S product solution. In the radio-trace, the principal peak at 8.59 min (93.2%) and the impurity at 9.48 min (5.5%) are visible. At the UV-trace, the peak at 9.32 min was assigned to cold PSMA I&S. The peak at 10.45 min was assigned to the formed dimer.

Radiochemical purity specifications with HPLC were adopted from the monographs for gallium-68 labelled peptides of the European Pharmacopoeia [12,13]. In the radiochromatogram, two minor regions next to the principal peak of [^99m^Tc]Tc-PSMA-I&S were observed (Figure 3). Experiments showed that the pH value of the reaction mixture could influence the percentage of region 2. For example, in the first batch prepared with 40 µL of 10 M NaOH, the percentage of the principal peak was 70.7%.In the three batches where 80 µL of NaOH were added, the percentage of the principal peak was 87.9 ± 0.5%. However, these batches did not meet the radiochemical purity specification of ≥91%. In three batches prepared with 120 µL of NaOH at pH 8.2, the percentage of the principal peak increased to 93.0 ± 0.3%, which corresponds to the radiochemical purity requirements. The results of this findings are summarized in Table 1.

### 2.3. Validation of the Automated Labelling of [^99m^Tc]Tc-PSMA-I&S

After exploring the reaction conditions, we did a full validation with the three batches where 120 µL of NaOH were added to the precursor solution. [^99m^Tc]Tc-PSMA-I&S was produced with a mean total activity of 1396 ± 270 MBq. The mean radiochemical yield was calculated based on the starting activity (2378 ± 450 MBq) and was 58.7 ± 1.5%.

The radiochemical purity of the compound was evaluated using HPLC and was 93.0 ± 0.3%. The amount of [^99m^Tc]TcO_4_^−^ evaluated using HPLC was 0.1 ± 0.03%.The colloidal [^99m^Tc]TcO_2_ was 0.3 ± 0.1% (TLC).

The stability of the [^99m^Tc]Tc-PSMA-I&S was confirmed 6 h prior to preparation using HPLC and TLC (92.8 ± 0.1%) and the amount of free [^99m^Tc]TcO_4_^−^ and [^99m^Tc]TcO2 was ≤0.5%.

The amount of Tc-PSMA-I&S, PSMA-I&S, and related substances in the product solution was evaluated using HPLC by comparing the area under the curve of the peaks found to an external standard of cold PSMA-I&S (5 µg/mL); it was 1.5 ± 0.2 µg/mL. The ligand PSMA-I&S owns a MAS3-group (2-mercaptoacetyl-ser-ser-ser) to specifically bind the technetium-99m (see Figure 1). While working with the unlabelled ligand, we noticed the appearance of a second peak (Rt = 10.5 min) beside PSMA-I&S (Rt = 9.3 min). It is probably the dimer formed through oxidation of the mercaptoacetyl group. Therefore, the dimer peak was also assigned to PSMA-I&S at the HPLC UV trace. Figure 3 shows a representative radio–HPLC chromatogram of a [^99m^Tc]Tc-PSMA-I&S product solution.

Post-release tests included the determination of the ethanol content and HEPES content as well as bacterial endotoxins and sterility testing. Table 2 summarizes quality criteria and the results of the 3 masterbatches of [^99m^Tc]Tc-PSMA-I&S.

## 3. Discussion

[^99m^Tc]Tc-PSMA-I&S was planned to be introduced at the Division of Nuclear Medicine in Graz as a potential diagnostic alternative to [^68^Ga]Ga-PSMA-11 PET. Unfortunately, no approved kit is available to prepare this tracer; therefore, we decided to synthesize the compound on the Scintomics GRP module according to relevant monographs of the European Pharmacopoeia [14,15] and current good radiopharmacy practice (cGRPP) guidelines of the EANM [16]. With the SCC developer software, we created an automated process, including the transfer of [^99m^Tc]TcO_4_^−^ from a V-shaped vial to the reactor and the purification of the compound via solid phase extraction (SPE).

The precursor was dissolved in 1 mL of HEPES buffer, which is an original part of the ABX reagent and hardware kit. We added a freshly prepared solution of the reducing agent SnCl_2_ and ascorbic acid to the precursor solution and used a 10 M NaOH solution for pH adjustment. We explored the optimum composition of the reaction solution in preliminary experiments. The pH value of the reaction solution turned out to be crucial for the radiochemical yield and radiochemical purity.

After optimizing the reaction conditions, we validated the labelling process by producing three consecutive master batches of [^99m^Tc]Tc-PSMA-I&S. The purification of the compound was successfully carried out with a Sep-Pak^®^ Light C18 cartridge. Only a minimal amount of activity remained on the cartridge after elution of the compound with 50% ethanol. Free [^99m^Tc]TcO_4_^−^ was almost completely removed, and less than 1% of colloidal [^99m^Tc]TcO_2_ was found in the product solution.

Within 40 min runtime of the automated process, we prepared up to 1.6 GBq [^99m^Tc]Tc-PSMA-I&S. A radiochemical yield of over 55% related to the starting activity and a radiochemical purity of > 91% was achieved. These results qualify this labelling process for the clinical application.

We adapted an HPLC method to analyse ^68^Ga-labelled peptides and validated it for this new compound. For TLC, the standard solvent for the quality control of ^68^Ga-peptides was used to evaluate the amount of reduced hydrolysed technetium-99m. The complete quality control of [^99m^Tc]Tc-PSMA-I&S is similar to the routine quality control of ^68^Ga-labelled peptides, and can be easily carried out by experienced personnel.

## 4. Materials and Methods

### 4.1. Radiolabelling and Purification of [^99m^Tc]Tc-PSMA-I&S

The radiolabeling was carried out on a Scintomics GRP 4 V module (Fürstenfeldbruck, Bavaria, Germany). The labelling sequence was programmed with the Scintomics developer software. The dedicated reagent and hardware kit (SC-01-H) and the cassettes for synthesis of ^68^Ga- peptides (SC-01) were purchased from ABX (Radeberg, Saxony, Germany). The configuration of the labelling cassettes included four modifications: A V-Vial with a perforable seal (DWK, Mainz, Germany) was assembled with two Sterican needles (B. Braun Melsungen AG, Melsungen, Germany). Using silicone tubing lines, the long needle (Ø 0.90 × 70 mm) was connected to valve 7 and the short needle (Ø 0.60 × 30 mm) was connected to valve 6. The position of the silicone tubing to the ventilation port of the reaction vessel was connected to valve 11. The connection to the N_2_ outlet was changed to valve 12. A detailed description of these changes is shown in Table 3.

The GMP grade precursor PSMA-I&S (40 µg, lyoprotected with 4 mg mannitol in 2 mL vials) was purchased from piChem (Raaba-Grambach, Austria). The specifications of the reagents used were in accordance with the European Pharmacopoeia. Tin (II) chloride dehydrate (SnCl_2_ × 2 H_2_O), ascorbic acid, and NaOH 10 M in H_2_O were purchased from Sigma-Aldrich (Saint Louis, MO, USA) and used without further purification. Hydrochloric acid 1 M (HCl) was purchased from Merck (Darmstadt, Germany) and diluted with water for injection (Fresenius Kabi AG, Graz, Austria) at 0.1 mol/L. A SnCl_2_/ascorbic acid solution was prepared by dissolving 20 mg SnCl_2_ × 2 H_2_O and 20 mg ascorbic acid in 10 mL of 0.1 M HCl. The precursor (40 µg of PSMA-I&S in 4 mg mannitol) was dissolved in 1 mL HEPES buffer (1.5 M, original part of the ABX reagent kit). Then, we added 50 µL of the SnCl_2_/ascorbic acid solution (2 mg/mL) and adjusted the pH of the precursor solution with 10 M NaOH. The reaction mixture was transferred with a 3 mL syringe into the reaction vial.

Sodium pertechnetate for injection ([^99m^Tc]TcO_4_^−^) was eluted from a Poltechnet ^99^Mo/^99m^Tc- Radionuclide generator purchased from POLATOM (Otwok, Poland). For the synthesis, the starting activity was transferred into the V-shaped vial.

### 4.2. Quality Control by HPLC

HPLC was performed on an Agilent 1260 series (Waldbronn, Baden-Wuerttemberg, Germany) equipped with a DAD UV detector (UV-VIS at λ = 220 nm) and a GABI star radiometric detector (Raytest, Straubenhardt, Germany). An ACE^®^3 C18 column (150 × 3.0 mm, Advanced Chromatography Technologies, Aberdeen, UK) was eluted by gradient elution (0.42 mL/min) with water/TFA 0.1% (solvent A) and ACN/TFA 0.1% (solvent B): Start 24% B; 3–12 min 40% B, 14–16 min 24% B. The total runtime was 30 min. The stock solution was prepared with 40 µg of the lyoprotected precursor in the 2 mL vial by diluting it with 1 mL of 0.01 M NaOH. The calibration standards were prepared by further diluting the stock solution with PBS. To analyse the chemical and radiochemical purity of PSMA-I&S, we validated this HPLC method according to the ICH Q2 (R1) guideline in the operating range of 1.0–10.0 µg/mL [17]. We evaluated a limit of quantification (LOQ) of 1 µg/mL within a linearity with a coefficient of correlation of 0.9996.

### 4.3. Quality Control by TLC

ITLC SG plates (Agilent, Waldbronn, Baden-Wuerttemberg, Germany) and the standard solvent for the quality control for ^68^Ga labelled peptides consist of a 1:1 solution of methanol and 1 M ammonium acetate. The radiolabelled compound [^99m^Tc]Tc-PSMA-I&S, as well as [^99m^Tc]TcO_4_^−^, moved to the front, while reduced hydrolysed [^99m^Tc]TcO_2_ remained at the start.

### 4.4. Evaluation of the pH Value

We determined the pH value of the reaction solutions and the product solutions by using PEHANON pH indicator strips (Macherey-Nagel, Düren, Germany).

### 4.5. Post Release Tests

Instead of a TLC limit test for the HEPES content according to the European pharmacopoeia [12,13], we used our validated HPLC method, modified from Antunes et al. [18]. A 150 × 4.6 mm XBridge C18 5 µm column (Waters, Milford, MA, USA) was used as a stationary phase, and 20 mM solution of ammonium formate (pH 8) as a mobile phase. The flow rate was 0.7 mL/min. The HEPES reference solution (40 µg/mL), the system suitability test with 2, 5-dihydroxybenzoic acid, and the samples were determined at UV λ = 195 nm.

For bacterial endotoxin testing, we used a quantitative kinetic chromogenic Limulus Amebocyte Lysate assay (Kinetic-QCL test kit, Lonza, Walkersville, MD, USA), and apyrogenic 96-well microplates (Corning, NY, USA). The measurements were performed at UV λ = 405 nm with a FLUOstar Omega microplate reader (BMG Labtech, Ortenberg, Germany) [19].

Accredited laboratory sites tested the ethanol content and sterility according to the European Pharmacopoeia.

## 5. Conclusions

An automated process for the preparation of [^99m^Tc]Tc-PSMA-I&S was developed on a Scintomics GRP synthesizer with respect to good manufacturing practice (GMP) and cGRPP. An advantage is the use of commercially available hardware and reagent kits intended for ^68^Ga-labelled peptides with only minor modifications. For successful preparations of [^99m^Tc]Tc-PSMA-I&S, the pH of the reaction mixture must be adjusted with the addition of NaOH. In summary, the presented synthesis, as well as the quality control, can be easily integrated into everyday clinical practice.

## Figures and Tables

**Table 1:** Reaction conditions and radiochemical purity evaluation of [^99m^Tc]Tc-PSMA-I&S.

	*n* = 1	*n* = 2	*n* = 3	*n* = 3
NaOH [mmol]	0	0.4	0.8	1.2
pH value of the reaction solution	5.5	7.2	7.8	8.2
Starting activity [MBq]	1954 (100%)	2383 (100%)	2457 ± 309 (100%)	2378 ± 450 (100%)
[^99m^Tc]Tc-PSMA-I&S (EOS) [MBq]	10 (0.5%)	1098 (46.1%)	1453 ± 193 (59.2 ± 4.1%)	1396 ± 270 (58.7 ± 1.5%)
Retained on Sep-Pak^®^ [MBq]	713 (36.5%)	151 (6.4%)	60 ± 27 (2.4 ± 0.9%)	23 ± 3 (1.0 ± 0.3%)
Residue in reaction vial [MBq]	327 (16.7%)	130 (5.5%)	55 ± 31 (2.2 ± 1.1%)	22 ± 2 (0.9 ± 0.1%)
Proportions of the peaks evaluated by HPLC				
[^99m^Tc]TcO_4_^−^ [%]	n.d.	0.09	0.1 ± 0.03	0.1 ± 0.01
Region 1 (impurity) [%]	n.d.	1.2	1.6 ± 0.3	1.2 ± 0.03
[^99m^Tc]Tc-PSMA-I&S [%]	n.d.	70.7	87.9 ± 0.5	93.0 ± 0.3
Region 2 (impurity) [%]	n.d.	28.0	10.5 ± 0.6	5.7 ± 0.3
Amount of reduced hydrolysed technetium-99m (TLC)				
[^99m^Tc]TcO_2_ [%]	n.d.	0.3	0.2 ± 0.1	0.3 ± 0.1

**Table 2:** Results of the quality control of 3 masterbatches of [^99m^Tc]Tc-PSMA-I&S.

Quality Control	Method	Criteria	Result (*n* = 3)
Appearance	visual inspection	clear and colourless	conforms
pH value	pH indicator strips	4–8	6.6
Radioactivity concentration	dose calibrator		82 ± 16 MBq/mL
Identity of [^99m^Tc]Tc-PSMA-I&S (comparison with reference)	HPLC	Rt = 8–12 min	conforms
Impurity reduced hydrolysed Technetium-99m	Radio–iTLC	≤3.0%	0.3 ± 0.1%
Free [^99m^Tc]TcO_4_^−^	Radio–HPLC	≤2.0%	0.1 ± 0.03%
Radiochemical purity of [^99m^Tc]Tc-PSMA-I&S	Radio–HPLC	≥91.0%	93.0 ± 0.3%
Tc-PSMA-I&S, PSMA-I&S and related substances	HPLC	≤2.4 µg/mL	1.5 ± 0.2 µg/mL
Unspecific impurities	HPLC	≤2.4 µg/mL	≤1 µg/mL
Ethanol content	gas chromatography	≤10.0% (*v*/*v*)	conforms
HEPES content	HPLC	≤40 µg/mL	4.4 ± 3.1 µg/mL
Bacterial endotoxins	LAL test	≤175 IU/V	conforms
Sterility	Ph. Eur.	sterile	conforms

**Table 3:** Summary of modifications of the ^68^Ga-. V = Vertical port.

	Position	Materials	Details
Modification 1	6 V	Silicone tubing to V-Vial (purge tube)	40 cm, 2 blue Luer male fittings, short needle (Ø 0.60 × 30 mm)
Modification 2	7 V	Silicone tubing to V-Vial (transfer tube)	40 cm, 2 white Luer male fittings, long needle (Ø 0.90 × 70 mm)
Modification 3	11 V	Silicone tubing to reaction vial (ventilation port)	Original part of the cassette
Modification 4	12 V	Silicone tubing to MFC	Original part of the cassette

## Data Availability

Data are contained within the article.
